# Efficacy of a multifactorial intervention on therapeutic adherence in patients with chronic obstructive pulmonary disease (COPD): a randomized controlled trial

**DOI:** 10.1186/1471-2466-14-70

**Published:** 2014-04-25

**Authors:** José Leiva-Fernández, Francisca Leiva-Fernández, Antonio García-Ruiz, Daniel Prados-Torres, Pilar Barnestein-Fonseca

**Affiliations:** 1 Primary Health Care Centre of Vélez-Sur, Health Area Málaga Este-Axarquía, Vélez Málaga, Málaga, Spain; 2 Multiprofesional Family and Community Medicine Teaching Unit of Primary Care Trust Málaga, Málaga, Spain; 3 Farmacoeconomy and SRI Unit, Farmacoeconomy and Clinical Therapeutic Department, Faculty of Medicine, Malaga University, Málaga, Spain

**Keywords:** Therapeutic adherence, COPD, Educational intervention

## Abstract

**Background:**

Therapeutic adherence of patients with chronic obstructive pulmonary disease (COPD) is poor. This study evaluated the effectiveness of a multifactorial intervention on improving the therapeutic adherence in chronic obstructive pulmonary disease (COPD) patients with scheduled inhalation therapy.

**Methods:**

The study design consisted of a randomised controlled trial in a primary care setting. 146 patients diagnosed with COPD were randomly allocated into two groups using the block randomisation technique. One-year follow-ups with three visits were performed. The *intervention* consisted of motivational aspects related to adherence (beliefs and behaviour) in the form of group and individual interviews, cognitive aspects in the form of information about the illness and skills in the form of training in inhalation techniques. Cognitive-emotional aspects and training in inhalation techniques were reinforced during all visits of the intervention group. The main outcome measure was adherence to the medication regimen. Therapeutic adherence was determined by the percentage of patients classified as good adherent as evaluated by dose or pill count.

**Results:**

Of the 146 participants (mean age 69.8 years, 91.8% males), 41.1% reported adherence (41.9% of the control group and 40.3% of the intervention group). When multifactorial intervention was applied, the reported adherence was 32.4% for the control group and 48.6% for the intervention group, which showed a statistically significant difference (*p* = 0.046). Number needed to treat is 6.37. In the intervention group, cognitive aspects increased by 23.7% and skilled performance of inhalation techniques increased by 66.4%. The factors related to adherence when multifactorial intervention was applied were the number of exacerbations (OR = 0.66), visits to health centre (OR = 0.93) and devices (OR = 2.4); illness severity (OR = 0.67), beta-2-adrenergic (OR = 0.16) and xantine (OR = 0.19) treatment; activity (OR = 1.03) and impact (OR = 1.03) scales of the Saint George Respiratory Questionnaire.

**Conclusion:**

Application of the multifactorial intervention designed for this study (COPD information, dose reminders, audio-visual material, motivational aspects and training in inhalation techniques) resulted in an improvement in therapeutic adherence in COPD patients with scheduled inhalation therapy.

**Trial registration:**

Current Controlled Trials ISRCTN18841601.

## Background

Chronic obstructive pulmonary disease (COPD) is characterised by airflow limitations that are not fully reversible. COPD is expected to become one of the major health challenges of the next few decades [1]. Prevalence surveys suggest that nearly one-quarter of adults aged 40 years and older have mild airflow obstruction [2-4]. COPD is presently the fourth leading cause of death, but the World Health Organization (WHO) predicts that it will become the third leading cause of death by 2030 [5], as mortality resulting from cardiac diseases and stroke decreased over the last 30 years, while that of COPD doubled over the same period [6].

As with all chronic diseases, therapy non-adherence in patients with COPD is a common problem and contributes to adverse health outcomes, reduced quality of life and increased healthcare expenditures [7]. According to the WHO, patient adherence to long-term therapy averages 50% [8]. Adherence rates in clinical trials may be as high as 70 to 90% [9-11], but in clinical practice, they range from 10 to 40% [12-14].

Several factors are related to adherence in COPD patients. Medication regimens are particularly vulnerable to adherence problems because of the chronic nature of the disease, the use of multiple medications (polypharmacy) and periods of clinical remission [15,16]. Selection of the medication and the delivery device usually depend on the efficacy of the different inhaled medications and devices, but these choices may be influenced by factors that are not clinically relevant, such as the ability of the patient to use the device, which also affects treatment adherence [15]. Moreover, patients often need to make important behavioural and lifestyle changes, which are also very important to treatment adherence [15].

Few studies exist on interventions to improve therapeutic adherence in COPD patients and other important aspects of disease management. Most of these existing studies have also involved asthmatic patients [17-22]. The studies have focused mainly on quantifying adherence and less on exploring the factors that affect non-adherence, although most of the authors highlight the need to work in motivational and cognitive ways to improve patient understanding of both their illness and treatment [22].

Specific studies that consider educational programs exclusively designed for COPD patients show significant improvement in patient management of the disease, including fewer exacerbations, decreases in the use of rescue medication and improved knowledge about inhaler use [19,21]. These programs include information about the illness, treatment, the importance of adherence, managing exacerbations, instructions on inhalation techniques and recommendations regarding the proper time to ask for a doctor’s advice. In some cases, counselling to cease smoking or referrals to independent programs are also included.

The main objective of this study was to evaluate the effectiveness of a multifactorial intervention (COPD information, dose reminders, audio-visual material, motivational aspects and training in inhalation techniques) on improving the therapeutic adherence in COPD patients with scheduled inhalation therapy after 1 year of follow-up with two reinforcement visits (3 and 6 months after intervention). The second objective was to describe the change in the functional status and health related quality of life and the possible modifying factors related to adherence when multifactorial intervention is applied.

## Methods

The study was approved by the by the Ethical Committees of the Primary Care Trust Málaga (01/03/07) and Axarquía (13/05/08) and by the Committee of Clinical Trials of the University Hospital Virgen de la Victoria, Málaga (30/11/07). The study protocol was reviewed by the Spanish Agency of Medicine and Health Products.

### Participants, design and setting

The study protocol has been published elsewhere [23]. In brief, we used a multicentre randomised controlled trial enrolled a sample consisting of 146 patients with COPD selected by a non-probabilistic consecutive sampling method. The inclusion criteria were as follows: confirmed COPD diagnosis registered in the patient’s clinical record, clinical assistance at primary care centres in the Malaga area, prescription of scheduled inhalation therapy and written informed consent. Patients were excluded if they had other respiratory conditions that are not included in the COPD definition (bronchiectasis, asthma or cystic fibrosis) or cognitive impairment problems registered in their clinical record (dementia, Alzheimer’s, Parkinson’s or cognitive decline).

The sample size was calculated to detect an adherence percentage difference between groups of 25%, with a statistical power of 80% and a confidence level of 95%, assuming a percentage of expected losses of 20%.

Patients were contacted by telephone using their health centre records and invited to participate; they then received an appointment at the health centre. At this first appointment (inclusion visit), patients were given more detailed information about the study, and if they agreed to participate, they signed the written consent form.

Subjects were divided randomly into two groups, a control group and an intervention group, using the block randomisation technique (blocks of four patients). All of the patients attended the same number of appointments. The follow-up for both groups consisted of three visits at 3, 6 and 12 months.

### Intervention

The intervention consisted of two activities, a group session at the beginning of the study and individual interventions during the follow-up visits. In both activities we applied a multifactorial educational strategy with three parts considered to be the most relevant aspects related to treatment adherence in patients with COPD [21]:

Part 1: Motivational aspects used to improve adherence. Focus groups were used to reveal patient experiences and points of view about their illness and treatment, including treatment adherence [24]. This session was videotaped with the patients’ formal consent for later analysis. Researchers analysed these videos to determine the main motivational aspects to focus on during the individual visits [25].

Part 2: Cognitive aspects related to treatment adherence. The intervention group received information about the disease so they could be more confident and more conscious about the importance of their daily treatment.

Part 3: Skills development involving training in inhalation techniques. The intervention group was trained in the use of their inhalers according to SEPAR (Sociedad Española de Neumología y Cirugía Torácica) guidelines [26], received explanations as to why a good technique is important, and practiced the proper technique with placebo inhalers.

Groups consisted of 6 to 9 patients. The group session lasted approximately 2 hours, including the patient’s reception, the distribution of materials, the description of the contents and the farewell. All group sessions were conducted by two professionals with special training in motivational techniques and in the use of inhaler devices. Audio-visual and written materials were used in parts 2 and 3 of the intervention (a leaflet describing the most relevant aspects of the disease and a scheme on inhalation techniques). The individual visits were conducted by the same professional for each patient.

The information used to plan the individual intervention employed during the follow-up visits was extracted from the group session by at least two researchers using qualitative methods [24].

### Outcomes definition and measurement

The primary outcome was adherence to (or compliance with) a medication regimen, which is generally defined as “the extent to which a person’s behaviour—taking medication, following a diet and/or executing lifestyle changes—corresponds with agreed recommendations from a health care provider” [27]. Therapeutic adherence represented the percentage of patients classified as good adherent as evaluated using dose or pill count. The measurement of doses or pill was made counting the number of doses register in the counter of device or considering the number of pills lacking in the blister packs of the prescribed medication.

The dose/pill count is the number of pills or doses taken divided by the number of pills or doses prescribed and then multiplied by 100 (expressed as a percentage) [28]. According to the recommendations in Sackett et al. [29], it is considered good adherence when the counting result is between 80% (20% of doses/pills missed) and 110% (the patient inhales 10% more doses/pills) of the dose/pill count prescribed.

The cognitive aspects were measured using the Batalla Test [30]; this test provides information about the patients’ knowledge of their illness. The test has three questions, adapted to COPD, and we was considered a good knowledge when the answers properly the complete questionnaire.

Patient’s skills at performing inhalations techniques were measured following SEPAR guidelines [26], using a schedule containing the main steps of correct technique.

The secondary outcomes that were measured included functional status by forced spirometry (following SEPAR guidelines [31]) and health-related quality of life as determined by the St. George respiratory questionnaire (SGRQ) [32] and the EuroQoL-5D questionnaire [33].

### Covariates

Factors considered to be associated with adherence were included as independent variables. General factors consist of age, educational level (low: no schooling or can only read and write) and co-morbidities. Disease-specific factors consist of time since diagnosis, smoking habits (smokers, non-smokers, numbers of cigarettes, years smoking), prescribed COPD treatment, number of devices, exacerbations and health care visits because of COPD (defined using patient health centre records).

### Statistical analysis

A descriptive statistical analysis was performed for all of the study variables. We calculated the mean, median and standard deviations for quantitative variables and the absolute and relative frequencies for qualitative variables.

We conducted several univariate analyses: between-group comparison at baseline, comparison between the initial sample and the final sample (impact of losses on sample structure) and exploration of the relationship between the primary outcome (adherence) and all of the independent variables at the end of the study. The chi-squared test or analysis of variance (ANOVA) was used.

The relative risk reduction (RRR), the absolute risk reduction (ARR) and the number needed to treat (NNT) were calculated.

Several logistic regression models and fixed- and random-effects estimations were used for the primary outcome [treatment adherence (yes/no)]. The intervention was considered the predictive variable, and the rest of the independent measures that were statistically significant during univariate analysis or that were considered clinically relevant because they were related to adherence in other studies [15,16,34-36] were considered the possible modifying factors.

To account for losses at the end of study, we built four partial models considering different independent variables and, at the end, we performed a final model to describe the modifying factors in our study. Model 1 was built using the sociodemographic profile (cohort, age and educational level). Model 2 included the utilisation of resources (cohort, number of general visits to the health care centre or because of COPD). Model 3 was built considering functional characteristics (cohort, spirometry pattern, severity level, FEV1 and FVC percentages, number of exacerbations and SGRQ scales). Model 4 included treatment (cohort, Beta-2 adrenergic agonist, inhaled and oral corticosteroids, s, xanthine treatment and the number of devices). The final model was built using cohort, age, educational level, HBP, the number of general visits to the health care centre, severity level, the number of exacerbations, the impact and activity scales of the SGRQ, Beta-2 adrenergic agonist, s, xanthine treatment and the number of devices.

The analysis was performed under an intention-to-treat procedure. We used a 5% significance level (f = 0.05) and the SPSS statistical package, version 15.0, to run the proposed analysis. Logit modelling was built using the Stata statistical package, version 11.1.

## Results

### Recruitment and follow-up

**Figure 1:**
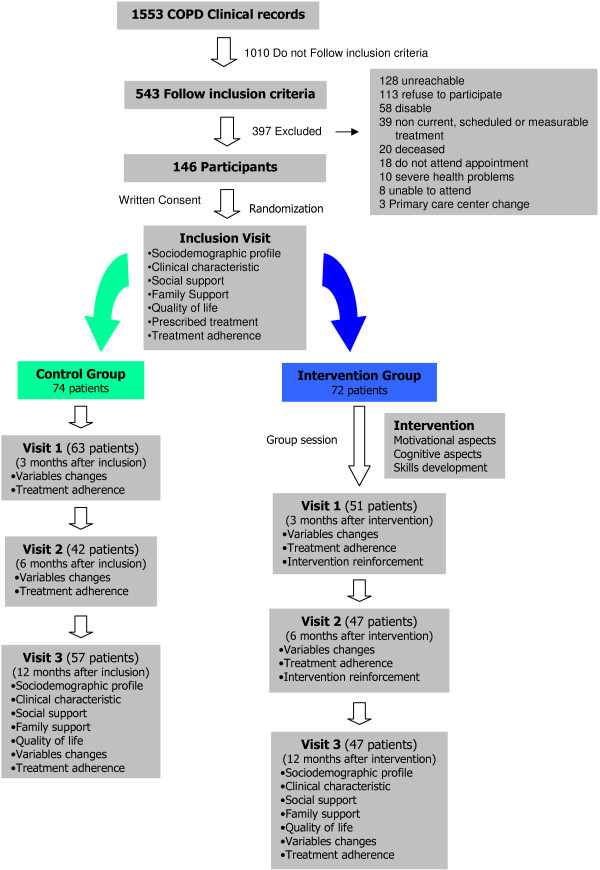
Study scheme.

A total of 1553 people were screened for inclusion visit in the study. Three hundred and ninety seven did not fulfil any of the inclusion criteria or had at least one of the exclusion criteria and were excluded from the study. Figure 1 shows the flow of patients in the study and reasons for exclusion.

### Patients’ baseline characteristics

The sample consisted of 146 patients with COPD. The patients were predominantly male (91.8%), with a mean age of 69.08 years (95% CI, 67.58-70.44 years), and 78.1% had a low educational level (4.1% had no studies, 29.5% were able to read and write, and 44.5% had a primary education) (Table 1).

**Table 1:** Baseline comparison between the study groups

**Variables (measure)**	**CG**	**IG**	***p***
Adherence (%)	41.9	40.3	0.843*
Sex (% males)	91.9%	91.7%	0.96*
Age (years)	68.59 (66.4-70.78)	69.57 (67.74-71.4)	0.509**
Low Educational Level	81%	75%	0.683*
Current Smoking (%)	33.8%	30.6%	0.676*
Smoking history (Pack-years)	58.67 (48.68-68.66)	67.07 (55.88-78.26)	0.275**
Cigarettes/day	21.32 (19.3-23.34)	19.45 (15.87-23.03)	0.683**
BMI (Kg/m^2^)	31.42 (27.47-35.37)	30.11 (29.06-31.16)	0.538**
Comorbidity			
• HBP (%)	40	58,3	0.032*
• DM (%)	25.7	38.9	0.088*
• OA (%)	33.8	29.2	0.548*
Diagnosis time (years)	4.27 (3.57-4.97)	4.61 (3.63-5.59)	0.585**
Pattern (%)			0.673*
• Obstructive	47.8	54.9	
• Restrictive	13	12.7	
• Mixed	39.1	32.4	
Severity (%)			0.630*
• Mild	34.8	40.6	
• Moderate	43,5	43,5	
• Severe	21.7	15.9	
Exacerbations per years	0.82 (0.59-1.05)	0.92 (0.67-1.17)	0.642**
Visits HC due COPD	1.56 (1.15-1.97)	2.04 (1.54-2.54)	0.162**
Treatment (%)			
• Anticholinergic	77	77.8	0.914*
• Beta-2 adrenergic	79.7	80.6	0.900*
• Inhaled corticosteroids	74.3	66.7	0.310*
• Xanthine	6.8	9.7	0.514*
Number of Devices (%)			0.913*
• 1	29.7%	30.6%	
• 2/more	70.3%	69.4%	
SGRQ (scores)			
Total	39.53 (35,42-43.64)	35.32 (31.35-39.29)	0.152**
• Activity	52.05 (48.12-57.98)	50.62 (45.83-55.4)	0.49**
• Symptoms	42.74 (38.14-47.34)	39.62 (35.21-44.03)	0.341**
• Impact	30.66 (26.45-34.87)	25.46 (21.41-29.51)	0.085**
EuroQoL5D-3 L (%without problem)			
• Mobility	54.1	59.7	0.489*
• Self-care	82.4	86.1	0.542*
• Perform main, family or leisure activities	77	80.6	0.601*
• Anxious or depressed	40.5	50	0.099*
• Pain or discomfort	64.9	70.8	0.207*

Quantitative variables: mean (CI 95%); Qualitative variables: %.

CG: control group; IG: intervention group; BMI: body mass index; HBP: high blood pressure; DM: diabetes mellitus; OA: osteoarthritis HC: health center; SGRQ: Saint George Respiratory Questionnaire.

*ji-schare test; **ANOVA test.

The mean body mass index was 30.78 kg/m^2^ (95% CI, 28.78-32.78). In terms of smoking, 93.8% of the patients had smoked, and 32.2% were active smokers, with a mean of 62.84 pack-years (95% CI, 55.34-70.34). Most patients suffered from at least one additional pathological condition, the most prevalent of which was high blood pressure (49.3%), followed by diabetes (32.2%) and osteoarthritis (31.5%). The patients had experienced at least one exacerbation over the last year, with a mean value of 0.87 exacerbations (95% CI, 0.68-1.06). Patients had consulted with the health centre because of COPD a mean of 1.8 times over the last year (95% CI, 1.47-2.13), which accounted for 26% of the total health visits.

The study included patients who had a COPD diagnosis in their clinical records, 91% of which were based on spirometric and clinical criteria. The mean time of COPD diagnosis was 4.43 years (95% CI, 3.56-5.3). The spirometry performed in the study revealed a mean FEV1 of 67.58% (95% CI, 64.58-71.08), with a predominant obstructive pattern (51.4%). Based on SEPAR [37] values, severity was distributed as follows: 37.7% mild stage, 43.5% moderate stage and 18.8% severe stage.

The major drugs prescribed for COPD were as follows: 80.1% of the patients were taking Beta-2 adrenergic agonists, 77.4% were taking anti-cholinergic drugs, and 70.5% had been prescribed inhaled corticosteroids. We also observed that 8.2% had a xanthine prescription, 11.6% were being treated with mucolytics, and 0.7% were being treated with oral corticosteroids. In most cases, patients were receiving combined therapy with more than one drug. The most frequent treatment (39.7%) was a 3-drug combination, normally consisting of an anti-cholinergic with a Beta-2 adrenergic agonist and an inhaled corticosteroid.

The health-related quality of life measured by the SGRQ in the total sample showed the following results according to its different scales: symptoms 41.2 (95% CI, 38.01-44.39), activity 51.86 (95% CI, 48.43-55.29), impact 28.1 (95% CI, 25.16-31.04) and total 37.47 (95% CI, 34.61-40.33).

According to the EuroQoL-5D-3 L scores in the total sample, less impairment was seen in mobility (56.8%); performance of main, family or leisure activities (78.8%); and self-care (84.2%). Additionally, 67.8% showed no anxiousness or depression, and 45.2% had no pain or discomfort.

In Table 1, we show the baseline comparison between the intervention and control groups; no significant differences were observed between groups except for high blood pressure (the intervention group had more hypertensive patients) (*p* = 0.032).

At the end of the follow-up period, the dropout rate was 28.8% (42 patients: 27 were excluded because they gave the inclusion criteria out, 16 of them did not attend the group session in intervention group; 6 did not attend the appointment; 4 refuse to follow in the study; 3 were unreachable and 2 deceased). We found differences in the spirometric pattern, there were less patients with obstructive pattern at the end of study (baseline: 68.6%; end of study: 33%; *p =* 0.017). The percentage of FEV1 (baseline: 67.83%; end of study: 60.38%; *p =* 0.006), FVC (baseline: 82.75%; end of study: 69.71%; *p =* 0.001), the number of exacerbations (baseline: 0.87; end of study: 0.43; *p =* 0.001), the number of general visits to the health centre (baseline: 6.93; end of study: 3.53; *p =* 0.001) or visits because of COPD (baseline: 1.8; end of study: 1; *p =* 0.001) were lower at the end of study and the oral corticosteroid prescription (baseline: 0.0068%; end of study: 0.046%; *p =* 0.039) was higher. No differences were found between patients lost to follow up in the intervention or in the control group.

### Intervention effectiveness

**Figure 2:**
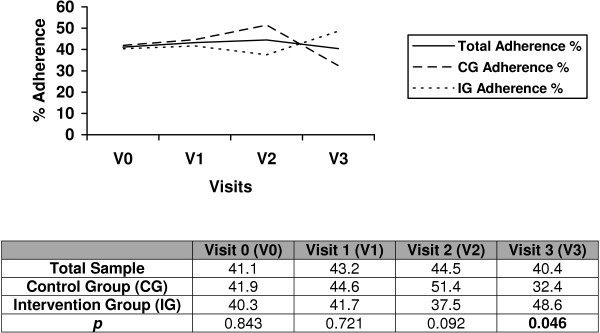
% percentage of adherence during follow-up measured by dose/pill count. Intention to treat analysis.

The change in adherence level according to dose/pill count in the total sample and in each group using per protocol analysis shows that the intervention group reached a rate of 79.5% for treatment adherence and that the control group reached a rate of 49% (*p* = 0.002). When we used intention-to-treat analysis (Figure 2), the difference between groups remained statistically significant (*p* = 0.046), and it became necessary to apply the intervention to 6 or 7 patients to obtain another adherent patient (Table 2).

**Table 2:** Intervention effectiveness on treatment adherence

**Parameters**	**APP**	**ITT**
	**Value (95% CI)**	**Value (95% CI)**
**Control**	0.489	0.324
**Interv**	0.795	0.481
**RR**	1.62 (1.35-1.93)	1.48 (1.07-2.04)
**RBI**	0.62	0.48
**ABI**	0.306 (0.216-0.396)	0.157 (0.007-0.307)
**NNT**	3.26 (2.52-4.62)	6.37 (3.25-142.8)

RR: Relative risk; RBI: Relative benefit increase; ABI: absolute benefit increase; NNT: number needed to treat; CI: Confidential interval. APP: analysis per protocol; ITT: Intention to treat analysis.

Relating to cognitive aspects, the intervention group showed an increase of 23.7% as measured by the Batalla test [30]. At the end of study, the difference between groups was statistically significant (*p* = 0.006) when the analysis per protocol was used, but it was not statistically significant with the intention-to-treat analysis.

**Figure 3:**
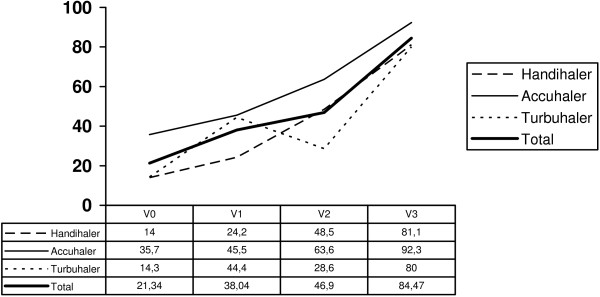
Percentage of patients who performs a correct inhalation technique per device after one year follow-up.

Patients’ skills at performing inhalation techniques were measured only in the intervention group, and there was better management of the inhalation devices at the end of the study (Figure 3).

### Factors related to treatment adherence

#### Univariate analyses

A significant difference was detected between adherence and the intervention applied (*p* = 0.046). There was no association with age, sex, educational level, smoking habits, comorbidity or time since diagnosis. Relating to functional status, an association was detected for spirometric pattern (adherent patients had an obstructive pattern 75.8% and no adherent patient had a restrictive pattern 68.8%; *p* = 0.009) and FVC percentage (adherent patients: 74.26%; no adherent patient 63.67%; *p* = 0.006), but not with severity or FEV1 percentage.

An association was found between adherence and the prescription of Beta-2 adrenergic agonists (adherent patients: 76.2%; no adherent patient 23.8%; *p* = 0.031), xanthine (adherent patients: 20%; no adherent patient 80%; *p* = 0,019), inhaled corticosteroids (adherent patients: 29.6%; no adherent patient 70.4%; *p* = 0,066) and mucolytics (adherent patients: 18.2%; no adherent patient 81.8%; *p* = 0,009).

There was no association with quality of life measurements and adherence. The total scale of the SGRQ was similar in both groups, but the scores on the symptom and activity scales were higher in non-adherent patients, while the scores on the impact scale were higher in adherent patients.

#### Multivariate analyses

After considering the univariate analyses, we performed the four partial fixed-effects logistic regression models described above. All of the models were adjusted by the presence of high blood pressure because we found a significant difference for this variable at baseline between the study groups.

The final model (Table 3) was built using a random-effects approach considering as predictive variables those variables that were included in the final model of the four partial models: cohort; age; educational level; prescription of Beta-2 adrenergic agonists, xanthine and mucolitycs; the number of different devices; severity; the number of exacerbations; the number of visits to the health care centre; and the activity and impact scales of the SGRQ. The final logit model obtained showed that adherence is favoured by the multifactorial intervention (OR = 1.88), a lower number of exacerbations (OR = 0.66), a lower number of visits to the health centre (OR = 0.93), a lower severity (OR = 0.677) and a lower number of devices (OR = 2.4). In terms of quality of life, adherence is favoured by a lower impairment of daily activities (OR = 0.978) and a higher disease impact (OR = 1.03). Beta-2 adrenergic agonist (OR = 0.16) and xanthine (OR = 0.199) treatments are associated with no adherence in our model. The rho coefficient in this model was 6.07×10^−6^ (*p* = 0.498).

**Table 3:** Treatment adherence random-effects logistic regression model

**Variables**	**OR**	**Std error**	***z***	***p***	**95% CI**
**Study Group**	1.887769	0.6012	1.99	0.046	1.01-3.52
**Age**	0.992041	0.0183	−0.43	0.666	0.95-1.02
**Educational level**	1.653566	0.5637	1.48	0.140	0.84-3.22
**Number of visit to HC**	0.938932	0.0332	−1.78	0.075	0.87-1.00
**Severity**	0.677282	0.1504	−1.75	0.079	0.43-1.04
**Number of exacerbation**	0.66481	0.1065	−2.55	0.011	0.48-0.91
**HBP**	0. 84349	0.2607	−0.55	0.582	0.46-1.54
**Beta2-adernergic**	0.16106	0.0814	−3.61	0.000	0.059-0.43
**Xanthine**	0.19933	0.1382	−2.33	0.020	0.05-0.77
**Mucolitys**	0.36196	0.1991	−1.85	0.065	0.12-1.06
**Number of devices**	2.4113	0.9696	2.19	0.029	1.09-5.30
**SGRQ (activity scale)**	0.97878	0.0120	−1.75	0.081	0.95-1.00
**SGRQ (impact scale)**	1.0310	0.0151	2.08	0.038	1.00-1.06
**Sigma_u**	0.0044	0.6345			6.0e-124-3.3e + 118
**Rho**	6.07e-06	0.0017			1.1e-247-1

Likelihood-ratio test of rho = 0: chibar2(01) = 1.6e-05 Prob > =chibar2 = 0.498.

## Discussion

The sociodemographic characteristics of our sample are similar to those in other studies that were performed in primary care settings [4,38-41]. COPD presents in older ages and predominates in males. Some previous studies have shown that the male sex is not a risk factor for COPD, and it appears that women could be more prone to COPD specifically because of tobacco exposure [42,43]. In our region, we retained a high prevalence in the male sex, most likely because of the smoking habits. Tobacco is considered one of the most important initial diagnostic characteristics for identifying chronic obstruction, and it is the major risk factor for COPD [44,45]. In our study, 34.2% of patients had a low educational level, which could contribute to poorer health education and greater difficulty in using health resources. Thus, we performed an educational intervention adapted to these patients using audio-visual material; we also determined that the questionnaires would be administered by the interviewer to help people who did not read or write fluently.

Our prescription profile is similar to other studies [46,47] and is concordant with national and international guidelines for the prescription of the major long-acting bronchodilator drugs [45,48]. The anti-cholinergic and Beta-2 adrenergic agonists are the most frequently prescribed drugs. Our subjects had a high prescription level of inhaled corticosteroids, particularly when taking into account that they had only mild to moderate severity.

The prevalence of adherence was 41%, while the observed levels of inadequate adherence were in line with other studies on COPD [15,27,38,41] and other chronic diseases [49]. Adherence to therapy in COPD is complex. Patients with COPD require adequate education on the disease process, comorbidities and the use of different medications and devices. They often need to make important behavioural and lifestyle changes as well.

### Intervention efficacy

No standard intervention exists to improve adherence. Haynes et al. [21] showed that there are many ways to perform interventions for adherence, but few studies exist about how to do it, possibly because of the complexity of this aspect. All of the effective interventions for long-term care were complex and included different combinations of more convenient care, information, reminders, self-monitoring, reinforcement, counselling, family therapy, psychological therapy, crisis intervention, telephone follow-up and supportive care. In COPD patients, existing studies on interventions to improve adherence are scarce, and many of them have been conducted with the inclusion of asthma patients [21]. The results are limited because the characteristics and responses to intervention are different for each disease [21]. Recent studies on adherence in COPD patients are centred on the description of factors that could have influenced it [15,16]. Specific studies that consider educational programs exclusively for COPD patients show significant improvement in patient management of the disease, fewer exacerbations, decreases in the use of rescue medications and improved knowledge about inhaler use [20,21].

The intervention designed in this study shows significant improvement in the therapeutic adherence of patients, confirming that multifactorial interventions are appropriated for patients with COPD. Looking at the data of visit 2, control shows a better adherence than intervention group, although at visit 3 the percentage of adherent patients is greater in the intervention group. Even though regression to mean might be a possible explanation for the increase rate of adherent in the control group, we think that the great amount of losses at visit 2 in this group, could explain also these results (most of the losses may have been the less motivated and adherent patients).

Our intervention focuses on three aspects: motivation, knowledge and skilled inhalation technique. Motivational aspects are related to patient beliefs and attitudes about their illness, their treatment and their physician. We emphasised these aspects because adherence includes a behavioural component and behaviour is slow and difficult to change [15,36]. New strategies based on shared decision-making appear to be the best approach to the behavioural component because treatment adherence includes a patient’s commitment to treatment, including the therapeutic regimen and the behavioural changes (smoking cessation, exercise) required to correctly manage their COPD. Khdour et al. [36] indicate that strategies aimed at improving COPD patient adherence must include the establishment of a personal relationship or partnership in which the patient feels able to discuss their fears, concerns and personal goals. In this regard, the motivational interview is very useful because the patients express their doubts and fears, and the doctor can act individually on each problem.

The second aspect of our intervention is the cognitive improvement related to COPD found in the intervention group, which is consistent with other studies concluding that COPD patients classified as adherent had greater understanding of their illness and their options for disease management [36]. Although the cognitive aspect could be related to educational level, no association was found in our patients. According to the literature, independently of educational level, instruction about the illness and its treatment is useful and has a significant impact on adherence [15].

The last aspect of our intervention concerns training in inhalation techniques. It is critical to evaluate whether patients are able to use their inhaler device correctly. Significant evidence shows that nearly 90% of patients with COPD incorrectly use their inhalers and that many of them display a technique that possibly delivers an inadequate dose [15,50]. The training program included in our intervention improved the percentage of patients who could perform a correct inhalation technique. This improvement was evident at the first follow-up visit because many of the study subjects had never been trained on inhalation techniques. Regular instruction, supervision, and follow-up on inhalation techniques in primary care are the responsibility of health professionals (physicians and nurses) [51]. Under real-life conditions, inappropriate inhaler use is common and strongly correlates with a lack of instruction by the caregiver [15,52]. Because the measurement of inhalation technique was performed only in the intervention group, we would have needed a control group to determine the precise effect of training; however, a 66.46% increase in the use of correct technique does not appear to be entirely random. We are currently conducting a clinical trial to test the efficacy of this training [53], after which we will be able to quantify the effect of intervention on inhalation techniques.

### Modifying factors related to adherence when multifactorial intervention is applied

In addition to receiving the designed multifactorial intervention, the models obtained from the multivariate analysis show that adherence is also related to the number of visits to the health centre; the number of exacerbations; disease severity; health status as measured by the activity and impact scales of the SGRQ; Beta-2 adrenergic agonist and xanthine prescriptions; and the number of devices.

The more adherent patients required fewer visits to the health centre and had fewer exacerbations, a lower number of devices, a lower level of severity, and a lower impact on daily activities, but with a higher disease impact. Beta-2 adrenergic agonist and xanthine treatments were associated with no adherence.

In this study, adherent patients with COPD had an incipient diagnosis of COPD [53] (mild severity and obstructive pattern). At this stage the illness had less of an impact on daily life activities (DLAs), possibly because of limited breathlessness. Patients often unconsciously moderated their activities to reduce the intensity of this distressing symptom. In general, COPD is characterised by a gradual deterioration in lung function aggravated by exacerbations [54]. Symptoms, functional status, and health-related quality of life (HRQoL) may follow different trajectories, and this discordance between functional and health outcomes results from a complex interaction of different variables, such as changes in health expectations, activity levels, and physical reconditioning. In fact, adherence might be more related to the patient’s beliefs about how he is feeling than to the spirometry results. Dyspnea and fatigue begin to have an impact on DLAs, and thus DLAs become limited but are not eliminated. Whether a DLA is eliminated depends on the relationship between the need or desirability of that activity and the difficulty in doing it because of the intensity of the associated symptoms. DLAs such as leisure activities are often the first activities to be eliminated, as they generally require greater effort and are not critical to daily life [55].

Adherent patients have better self-management, which is related to better knowledge about their illness and inhalation techniques [15,16,32], and in our model, more adherent patients had fewer exacerbations and required fewer visits to the health centre.

Related to treatment, the prescription of Beta-2 adrenergic agonists and xanthine was associated with no adherence, and having fewer devices was associated with adherence. Thus, polypharmacy is directly related to non-adherence [15,16,34]. Moreover, Beta-2 adrenergic agonists allow patients to end symptoms quickly, causing them to intake more doses than are prescribed, which consequently leads to non-adherent status.

### Strengths and limitations

This study has several strengths. First, it is a randomised controlled trial, regarded as the gold standard for assessing the effectiveness of treatments. The random allocation of patients reduced the probability that bias would affect the outcomes [56]. Second, we standardised the intervention. The working plan was structured in an exhaustive manner, and the intervention was performed by two professionals trained in communication, knowledge of COPD and the inhalation techniques associated with the different devices used by these patients. Furthermore, we designed a manual for the researchers that explained the working plan, the different parts of the intervention, the protocol scheme outlining what to measure each time and the details to assess each variable included in the study. Therefore, the procedure can be replicated elsewhere. Third, although the measure of adherence was performed using an indirect method, the dose/pill count has been used in outpatient practices as the reference standard to define the diagnostic validity of self-reported methods [37,57,58]. The main disadvantage of the dose/pill count is that it assumes that every missing dose/pill from the package has been taken by the patient; thus, it could overestimate the prevalence of treatment adherence. However, this method is simple, readily available and less expensive than other methods. No perfect gold standard exists owing to the nature of the process that we are studying, and thus, we assume the imperfect gold standard bias is present in our study [59]. Fourth, patient beliefs, experiences, and behaviours with regard to both the disease and the treatment were found to be more powerful predictors of medication adherence than sociodemographic and clinical factors in patients with COPD [15,22,35]. We therefore explored why the recommended therapeutic regimen was not completed in the first part of the intervention (group session) and in the individual visits. In the group session, qualitative methods such as the focus group technique were used to explore the beliefs and attitudes of the patients, while in the individual visits, this information was used to design individual interviews to focus on motivational aspects to improve treatment adherence, using techniques from the motivational interview. Thus, the behavioural changes would be more permanent.

This study also has several limitations. The first limitation is the selection bias resulting from the missing data. To diminish this bias, we applied several strategies: an increase of 20% in the sample size (expected losses), three phone calls on different days and at different times for unreachable patients and additional appointments for the patients who did not attend the clinic visits (three different appointments). Despite this, we had a dropout percentage that was higher than expected. We analysed the similarities between the initial sample and the final sample, and several differences were found (spirometric pattern and the percentage of FEV1 and FVC). However, these differences were related to illness progression rather than to adherence. The loss of lung function appears to be accelerated and therefore more relevant in the early stages of COPD [60,61]. To confirm this assumption, we analysed the patients who attended the last visit and found a change in the spirometric pattern from an obstructive to a mixed pattern, an increase in the severity and a decline in FEV1 and FVC scores between the first and last visits. This progression to a more severe stage of the disease could explain the higher prescription level of oral corticosteroids in the final sample. The differences found in the number of exacerbations, the number of visits to a primary care centre and the number of visits because of COPD were statistically significant in the intervention group (the number is lower at the end of study). This finding could be explained by the intervention design, which may have contributed to an improvement in the self-management of COPD in these patients as a result of better knowledge about their illness and inhalation techniques. The nature of the applied intervention requires that patients determine their own lack of knowledge through the motivational interview. In the control group, only the number of visits to a primary care centre was different between baseline and the final visit (lower at the end of study), possibly because of the therapeutic effect (Hawthorne effect) of the follow-up visits. Another possible bias is the restrictive criteria that were used for patient selection. Patients included in the study had to have prescriptions for drugs whose doses could be counted, thus affecting the ability to generalise the results. However, in previous studies [38], our group has established a strategy based on the use of indirect methods to measure adherence in patients with devices that cannot be counted.

## Conclusion

Non-adherence is a significant health problem around the world because it contributes to adverse health outcomes, reduced quality of life and increased healthcare expenditures. However, this is an aspect of therapy that clinical practice guidelines do not emphasise as a prior step to adequate treatment. Our data show that the use of educational intervention can contribute to adherence in COPD patients and can allow us to better understand the complex concept of adherence. We must, however, take into account that adherence requires a behavioural change and that this aspect is related to individual interests and expectations, meaning that patients must be managed individually.

The findings of this randomised controlled trial show that a multifactorial intervention (COPD information, dose reminders, audio-visual material, motivational aspects and training in inhalation techniques) in COPD patients is associated with an increased benefit of 48% in the adherence to prescribed treatment. The intervention improved in a 14.8% the percentage of adherent patients comparing with no intervention, but we have to consider the intervention is complex and time-consuming. From the point of view of clinical significance it could be necessary to conduct a cost-effectiveness analysis of these results. Nevertheless, the results remained robust after controlling for potential confounder factors and losses and there are promising results in relation to inhalation techniques that should be consider for future research. Considering the magnitude of this health problem, physicians and nurses should consider the opportunity to intervene with these patients to ensure better health outcomes.

## Competing interests

The authors declare that they have no competing interests.

## Authors’ contributions

JLF is the main research fellow, he has been involved in the design of the study and he has participated in reviewing the manuscript. DPT has been involved in the design of the study, and he has participated in reviewing the manuscript. AGR has been involved in the design of the study and he has participated in reviewing the manuscript. FLF has been involved in drafting the manuscript and writing it. She has participated in the design of the study and the intervention. PBF has been involved in drafting the manuscript and writing it. She has participated in the design of the study and the intervention. All authors read and approved the final manuscripts.

## Pre-publication history

The pre-publication history for this paper can be accessed here:

http://www.biomedcentral.com/1471-2466/14/70/prepub
